# Nutlin‐3a selects for cells harbouring *TP* *53* mutations

**DOI:** 10.1002/ijc.30504

**Published:** 2016-11-22

**Authors:** Jill E. Kucab, Monica Hollstein, Volker M. Arlt, David H. Phillips

**Affiliations:** ^1^ King's College London, Analytical and Environmental Sciences Division, MRC‐PHE Centre for Environment & HealthLondonUnited Kingdom; ^2^ German Cancer Research Center (Deutsches Krebsforschungszentrum), Division of Genetic Alterations in CarcinogenesisHeidelbergGermany; ^3^ University of Leeds, Faculty of Medicine and HealthLeedsUnited Kingdom

**Keywords:** TP53, mutation, Nutlin‐3a, Hupki, immortalisation

## Abstract

*TP53* mutations occur in half of all human tumours. Mutagen‐induced or spontaneous *TP53* mutagenesis can be studied *in vitro* using the human *TP53* knock‐in (Hupki) mouse embryo fibroblast (HUF) immortalisation assay (HIMA). *TP53* mutations arise in up to 30% of mutagen‐treated, immortalised HUFs; however, mutants are not identified until *TP53* sequence analysis following immortalisation (2–5 months) and much effort is expended maintaining *TP53*‐WT cultures. In order to improve the selectivity of the HIMA for HUFs harbouring *TP53* mutations, we explored the use of Nutlin‐3a, an MDM2 inhibitor that leads to stabilisation and activation of wild‐type (WT) p53. First, we treated previously established immortal HUF lines carrying WT or mutated *TP53* with Nutlin‐3a to examine the effect on cell growth and p53 activation. Nutlin‐3a induced the p53 pathway in *TP53*‐WT HUFs and inhibited cell growth, whereas most *TP53*‐mutated HUFs were resistant to Nutlin‐3a. We then assessed whether Nutlin‐3a treatment could discriminate between *TP53*‐WT and *TP53*‐mutated cells during the HIMA (*n = 7*2 cultures). As immortal clones emerged from senescent cultures, each was treated with 10 µM Nutlin‐3a for 5 days and observed for sensitivity or resistance. *TP53* was subsequently sequenced from all immortalised clones. We found that all Nutlin‐3a‐resistant clones harboured *TP53* mutations, which were diverse in position and functional impact, while all but one of the Nutlin‐3a‐sensitive clones were *TP53*‐WT. These data suggest that including a Nutlin‐3a counter‐screen significantly improves the specificity and efficiency of the HIMA, whereby *TP53*‐mutated clones are selected prior to sequencing and *TP53*‐WT clones can be discarded.

## Abstract 2

**What's new?**

Because half of human tumors carry mutations in the *TP53* tumor suppressor gene, a mammalian cell culture system reproducing the diverse mutations seen in patients is necessary to properly study functional impact. Here the authors report a significant improvement to an existing model system using fibroblasts from human *TP53* knock‐in mice (HIMA). They show that treating cells with Nutlin‐3a, an inhibitor of the destabilizing cofactor MDM2, efficiently selects for the growth of *TP53*‐mutated immortalized cells, while selecting against *TP53*‐wild type cells, thus increasing specificity and efficiency of *TP53* mutagenesis.

**Abbreviations**
AA: aristolochic acid
BaP: benzo[*a*]pyrene
HIMA: HUF immortalisation assay
HUF: Hupki mouse embryo fibroblast
Hupki: human *TP53* knock‐in
MEF: mouse embryo fibroblast
UV: ultraviolet

The transcription factor p53 plays a vital role in the cellular response to stresses such as DNA damage. Depending on the severity of the stress, p53 can direct a cell toward cell cycle arrest, DNA repair or apoptosis by regulating the transcription of an array of genes.^1^ By preventing the growth of stressed or damaged cells, p53 acts as a key tumour suppressor. The gene encoding p53, *TP53*, is mutated in over half of all cancers.^2^ The majority of *TP53* mutations in cancer are missense and occur in the DNA‐binding domain coding region (exons 5–8). Typically, *TP53*‐mutated cells accumulate excessive levels of mutant protein that is unable to transcriptionally transactivate canonical p53 target genes and may possess new, gain‐of‐function activities.^3^ A comprehensive study of the transactivational capability of p53 mutants, representing all possible amino acid substitutions caused by point mutation, demonstrated that human tumour‐associated mutations are strongly correlated with inactivation of p53 function.^4^

Analysis of *TP53* mutations detected in human tumours, which are diverse in type, position and functional impact, has established correlations between specific mutation signatures and exposure to certain environmental mutagens (*e.g*. C > T and CC > TT mutations in head and neck squamous carcinomas, associated with ultraviolet (UV)‐radiation exposure; G > T mutations in smokers' lung cancers, associated with exposure to tobacco carcinogens, such as benzo[*a*]pyrene (BaP); A > T mutations in urothelial carcinomas, associated with exposure to aristolochic acid (AA).^2^ Some of these signatures have been replicated experimentally using embryo fibroblasts from the partial human *TP53* knock‐in (Hupki) mouse, in which exons 4–9 of human *TP53* replace the corresponding exons of murine *Trp53*.^5^, ^6^, ^7^

The Hupki mouse embryo fibroblast (HUF) immortalisation assay (or HIMA) was designed to generate and select for *TP53* mutations in a mammalian cell context, taking advantage of the fact that mutation or loss of *TP53* is a key mechanism enabling the immortalisation of HUFs. Mutations may be carcinogen‐induced or arise spontaneously and can be compared to the *TP53* mutation spectra found in human tumours to explore potential links with cancer aetiology. For the HIMA, cultures of primary HUFs are first treated with a carcinogen of interest. Treated HUFs, along with untreated control cultures, are then serially passaged according to a modified 3T3 protocol.^8^, ^9^ The majority of HUFs will undergo p53‐dependent senescent growth arrest, due to the sensitivity of mouse cells to atmospheric oxygen levels in standard cell culture (20%). HUFs that have accumulated mutations (*e.g*. in *TP53*) that permit senescence bypass will continue to proliferate as clonal populations and ultimately become established into immortalised cell lines. This selection process takes between 2 and 5 months, as some immortal clones emerge relatively quickly from senescent cultures, whereas other clones take longer to develop. DNA from the immortalised HUFs is then sequenced to identify *TP53* mutations. Previous studies have detected *TP53* mutations in up to 30% of mutagen‐treated cultures or 5 to 20% of spontaneously immortalised cultures.^10^, ^11^, ^12^, ^13^ The remaining cultures are *TP53*‐WT and likely harbour alterations in other genes associated with senescence‐bypass.^14^, ^15^

Because the HIMA mutant selection process (*i.e*. senescence bypass) is not fully specific for *TP53*‐mutated cells, the majority of the effort of the assay is expended maintaining *TP53*‐wild‐type (WT) cultures. An additional selection procedure to distinguish between cells harbouring WT or mutated *TP53* would greatly improve the efficiency of the assay. Such a selection step would ideally inhibit growth of HUFs containing WT *TP53* while permitting growth of *TP53*‐mutated cells. Pharmacological activation of p53 to arrest or kill tumour cells retaining WT *TP53* is currently an active field of research in cancer therapeutics.^16^ One emergent strategy is to disrupt the binding of p53 to its negative regulator MDM2. MDM2 inhibits p53 by: (*i*) ubiquitinating p53 to promote its proteasomal degradation;^17^ (*ii*) binding to the N‐terminal activation domain of p53, masking its ability to activate transcription;^18^ and (*iii*) participating in the nuclear export of p53.^19^ In turn, p53 regulates *MDM2* expression at the level of transcription as part of an autoregulatory feedback loop.^20^

Nutlin‐3a, a *cis*‐imidazoline analogue, binds the p53‐binding pocket of MDM2, inhibiting the interaction of MDM2 and p53 and resulting in p53 stabilisation and activation.^21^ Nutlin‐3a is non‐genotoxic, thus endogenous cellular stress may initiate activation of stabilised p53.^22^ Treatment of cells with Nutlin‐3a can induce the expression of p53 target genes (*e.g. p21^WAF1/Cip1^* and *MDM2*) and has been shown to induce arrest or apoptosis of cancer cells expressing WT p53.^23^, ^24^ Crucially, Nutlin‐3a specifically induced senescence of mouse embryo fibroblasts (MEFs) with WT *Trp53* but did not affect the growth of *Trp53*‐null MEFs.^22^ MEFs deficient in p53 pathway components *p19/ARF* or *p21^WAF1/Cip1^* retain Nutlin‐3a sensitivity.

We hypothesised that Nutlin‐3a could be applied to the HIMA as a counter‐screen following senescence bypass to discriminate between immortal HUF clones containing WT *TP53* and those with mutation or loss of *TP53*. We first examined the effect of Nutlin‐3a treatment on cell growth and p53 pathway activation using primary HUFs and previously established immortal HUF cell lines with and without mutated *TP53*. We then determined whether sensitivity or resistance to Nutlin‐3a treatment of immortal HUF clones, soon after their emergence from senescent cultures, was predictive of WT or mutated *TP53* upon sequence analysis of the DNA. We report that Nutlin‐3a can indeed specifically select for the growth of HUF clones in which *TP53* is mutated and that the majority of mutants can be identified within 2.5 months of initiating the HIMA.

## Material and Methods

### HUF cultures

Primary and immortalised Xpa‐WT and Xpa‐Null HUFs were derived from embryos of inter‐crossed *Hupki^+/+^;Xpa* ^+/−^ mice as described previously.^25^ HUFs were cultured in growth medium (Dulbecco's modified medium (Invitrogen #31966–021) supplemented with 10% fetal bovine serum (Invitrogen #26140–079) and 100 U/mL penicillin and streptomycin (Invitrogen #15140–130)) at 37°C/5% CO_2_ in either 3% O_2_ (primary HUFs) or 20% O_2_ (immortalised HUFs), adjusted using an incubator fitted with an oxygen sensor and a nitrogen source. For passaging cells were detached with 0.05% trypsin‐EDTA (Invitrogen #25300–062), suspended in growth media and reseeded at the desired dilution or cell number.

### HUF immortalisation assay (HIMA)

Xpa‐WT and Xpa‐Null primary HUFs were immortalised following treatment with 3‐nitrobenzanthrone (3‐NBA) or 0.1% DMSO control as described previously.^25^ Cell lines were named according to Xpa status (XW: Xpa‐WT, XN: Xpa‐Null) and treatment (C: control, 3N: 3‐NBA), followed by the clone number. A panel of immortal clones generated after treatment ± 1 µM 3‐NBA for 2 × 48 hr, including nine *TP53*‐mutants and five clones retaining WT *TP53* (Supporting Information Table 1), were used for an initial assessment of HUF responses to Nutlin‐3a. To assess a Nutlin‐3a counter‐screen in parallel to a HIMA, a further 36 cultures of Xpa‐WT or Xpa‐Null primary HUFs were treated with 1 µM 3‐NBA for 1 × 48 hr and passaged until immortalised clones emerged from the senescent cultures. Each clone was subjected to a counter‐screen with Nutlin‐3a as described below and continually passaged until immortalised cell lines were established (≥12 passages).

### Treatment with Nutlin‐3a

Nutlin‐3a (Cayman Chemicals #18585) was dissolved in DMSO to 10 or 20 mM and stored in aliquots at −20°C. For cell treatment, Nutlin‐3a was diluted in complete growth medium to final concentrations up to 10 or 20 µM (0.1% DMSO).

### Nutlin‐3a counter‐screen of HUFs during the HIMA

A Nutlin‐3a counter‐screen was conducted on clones from the HIMA described above. As proliferating clones emerged from senescent cultures (1–3 months after initiating the immortalisation assay; passage 6–10), each clone was split to two wells of a six‐well plate (note that the time required for senescence bypass and the subsequent doubling rate was variable between clones). One well was treated with 10 µM Nutlin‐3a while the other was left untreated and maintained according to the assay described by Kucab *et al*.^25^ After 5 days, Nutlin‐3a‐treated cells were visually inspected under the microscope to determine whether the culture was resistant (similar morphology and growth rate compared to untreated wells), sensitive (all cells observed to be growth‐arrested, possibly enlarged), or exhibited a mixed response (both sensitive, growth‐arrested cells and resistant, proliferating cells present).

#### Cultures with a mixed response to Nutlin‐3a

After treatment, Nutlin‐3a‐containing medium was removed from the cells and replaced with normal growth medium. When the Nutlin‐3a‐resistant population reached 60–80% confluency, cultures were expanded from six‐well plates, first to 25‐cm^2^ flasks, then to 75‐cm^2^ flasks and then diluted 1:20 at least once before further analysis. Nutlin‐3a‐resistant cultures were designated with an “R” following the name of the parental clone.

#### Nutlin‐3a‐sensitive cultures

After treatment, Nutlin‐3a‐containing medium was removed from the cells and replaced with normal growth medium. Cultures were visually inspected for recovery of proliferation over the next 7 days; those that rapidly recovered (≤3 days) were expanded and diluted as described above for Nutlin‐3a‐resistant cultures. Sensitive cultures that recovered from Nutlin‐3a treatment were designated with “rec” following the name of the parental clone.

### Isolation of single cell clones from immortalised HUF cell lines

Dilutions of immortal cell lines XW‐3N‐14/‐14R and XW‐3N‐15/‐15R were prepared at 2.5 or 5.0 cells/mL in growth medium and seeded onto 96‐well plates (200 µL/well). The medium was changed every 3–5 days for up to 2 weeks until single cell clones (SCCs) were established (∼50–100 cells per well). SCCs from 6–10 wells per cell line were passaged to 24‐well plates and then expanded progressively into larger vessels (up to 75‐cm^2^ flask) before further analysis.

### Cell growth/survival assay

Survival following treatment with Nutlin‐3a was assessed by crystal violet staining. Cells were seeded at 0.75–1.5 × 10^3^ cells/cm^2^ on 96‐well plates and treated the following day with Nutlin‐3a diluted in growth medium up to 10 µM (0.1% DMSO; five replicate wells per condition). Following treatment, cells were rinsed with PBS, stained for 15 min with 0.1% (w/v) crystal violet (Sigma #C3886) in 10% ethanol, washed with PBS and air‐dried. The dye retained by surviving cells was solubilised in 100 µL of 50% ethanol per well and A_595nm_ was determined using a plate reader. Data are presented as the percentage of A_595nm_ in Nutlin‐3a‐treated cells relative to that of control cells and are representative of at least two independent experiments.

### Western blotting

Cells were treated with Nutlin‐3a at 60–80% confluence for up to 24 hr, washed with PBS and lysed (62.5 mM Tris [pH 6.8], 1 mM EDTA, 2% SDS, 10% glycerol, 1X Halt™ Protease Inhibitor Cocktail (#1860932 Thermo Scientific, UK)). β‐Mercaptoethanol (0.1% v/v) and bromophenol blue (0.01% w/v) were added to each lysate prior to denaturation at 95°C for 5 min. Equal amounts of protein (10–20 µg) were separated by SDS‐PAGE on 4–12% Bis‐Tris gels (NuPAGE; #NP0336 Invitrogen) and transferred to nitrocellulose membranes. Membranes were incubated with primary antibody (anti‐p53 (#NCL‐p53‐CM5 Leica Microsystems; 1:1,000), anti‐p21 (#556431 BD Pharmingen; 1:2,000), anti‐Mdm2 (#ab16895 Abcam; 1:400), and anti‐Gapdh (#MAB374 Millipore, 1:25,000)) followed by species‐specific horseradish peroxidase‐conjugated secondary antibody (Bio‐Rad) and bands were detected by chemiluminescence.^26^

### *TP53* mutation analysis

DNA was extracted from cells using the Gentra Puregene Cell Kit B (Qiagen, #158745), according to the manufacturer's instructions. Mutations in human sequences of *TP53* (exons 4–9, and flanking splice sites) from DNA were detected as described recently.^10^, ^25^

## Results

### Nutlin‐3a selectively inhibits the growth of primary and immortal HUFs with WT *TP53*

**Figure 1:**
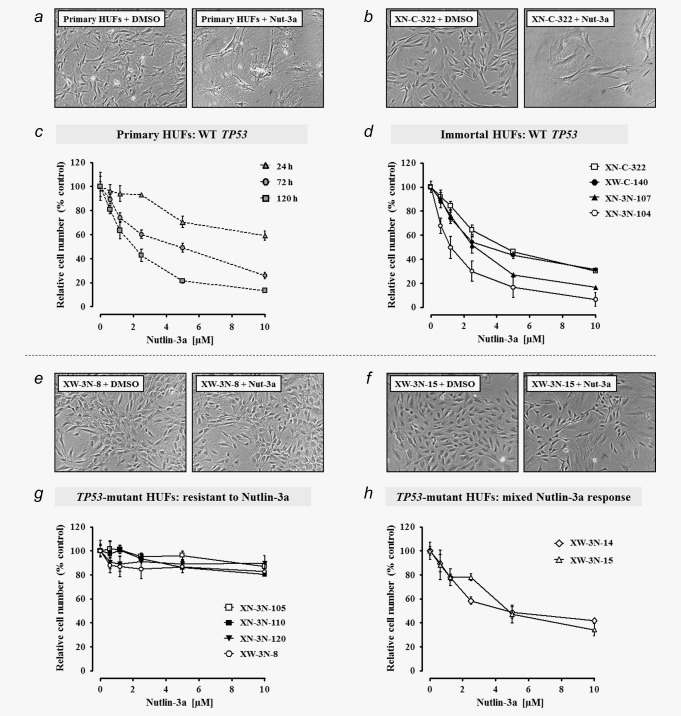
Nutlin‐3a inhibits the growth of *TP53*‐WT primary and immortalised HUFs, while *TP53*‐mutated cell lines exhibit resistance. Cells were treated ± Nutlin‐3a (up to 10 µM) for up to 5 days. Cells treated with 0.1% DMSO only served as control. Photomicrographs (100×) of (*a*) primary, (*b*) immortal *TP53*‐WT (XN‐C‐322), (*e*), immortal, Nutlin‐3a‐resistant *TP53*‐MUT (XW‐3N‐8) and (*f*) immortal, partially Nutlin‐3a‐resistant *TP53*‐MUT (XW‐3N‐15) HUFs treated ± 10 μM Nutlin‐3a for 5 days. (*c*, *d*, *g* and *h*) Relative cell number was determined by crystal violet staining and mean values are shown as % control (0.1% DMSO) ± SD of five replicate wells. Data are representative of at least two experiments.

It was previously shown that Nutlin‐3a treatment (5–10 µM for up to 1 week) inhibited the growth of primary p53‐WT MEFs, while p53‐Null MEFs were resistant.^22^ We therefore investigated whether HUFs with WT *TP53* would also be sensitive to Nutlin‐3a treatment, and whether HUFs carrying mutated (*i.e*. non‐functional) *TP53* would be resistant, using primary HUFs and a panel of immortal HUF cell lines with WT or mutated *TP53* generated previously (Fig. 1; Table 1).^25^

Following treatment with up to 10 µM Nutlin‐3a for 24, 72, or 120 hr, a dose‐dependent decrease in the growth of primary HUFs was observed, with maximal inhibition (∼85%) occurring in cells treated for 5 days (Fig. 1 *c*). The growth of Xpa‐WT and Xpa‐Null HUFs was inhibited to a similar extent (data not shown for Xpa‐Null cells). Cells treated with 10 µM Nutlin‐3a were significantly enlarged and flattened (Fig. 1 *a*). This morphology is similar to that of primary HUFs that undergo senescence after prolonged culture at 20% O_2_. We then assessed the growth of four immortal HUF cell lines with WT *TP53* (XN‐C‐322, XW‐C‐140, XN‐3N‐104, XN‐3N‐107) treated with up to 10 µM Nutlin‐3a for 5 days. As observed for primary HUFs, the growth of immortal HUFs with WT *TP53* was strongly inhibited by Nutlin‐3a, ranging from 70–95% inhibition at 10 µM (Fig. 1 *d*). The Nutlin‐3a‐treated immortal cells with WT *TP53* were enlarged and flattened (Fig. 1 *b*), similar to the morphology of Nutlin‐3a‐treated primary HUFs.

Next, we examined the growth of nine immortal HUF cell lines with MUT *TP53* in the presence of Nutlin‐3a. Each cell line was initially treated with 10 µM Nutlin‐3a (or 0.1% DMSO) for 5 days and examined under the microscope. Growth inhibition was assessed qualitatively (*i.e*. resistant, sensitive or mixed response; Table 1). Representative examples of Nutlin‐3a‐treated *TP53*‐MUT cells are shown in Figures 1e and 1*f*. With the exception of two lines, XW‐3N‐14 and XW‐3N‐15, *TP53*‐MUT HUFs were completely resistant to the growth‐inhibiting effects of Nutlin‐3a and maintained a morphology similar to that of untreated cells (Table 1 and Fig. 1 *e*). Interestingly, XW‐3N‐14 and XW‐3N‐15 exhibited a mixed response to Nutlin‐3a, containing both sensitive and resistant cells; clonal regions of proliferating cells were surrounded by enlarged, arrested cells (Fig. 1 *f*). We assessed further the effect of Nutlin‐3a on the growth of *TP53*‐MUT HUFs quantitatively, finding a slight decrease (10–20%) in cell growth at 10 µM for resistant mutants and a marked inhibition (60–70%) for mixed‐response mutants (Figs. 1 *g* and 1*h*).

**Table 1:** The effect of Nutlin‐3a on cell growth and p53 activity in *TP53*‐mutant HUF cell lines established A) prior to and B) in parallel to a Nutlin‐3a counter‐screen

	*TP53* mutation status		Response of HUF cell line to Nutlin‐3a			p53 mutant function (other studies)	
HUF cell line ID^a^	Mutation	Zygosity	Growth inhibition (5 days)	Protein induction (24 hr)	Activity^b^	Dominant negative
				p21			Mdm2
**A) Established prior to a Nutlin‐3a counter-screen**							
XW‐3N‐7	Y236C	Homo‐/hemi‐	Resistant	–	–	NF	N/A
XW‐3N‐8	H179N	Homo‐/hemi‐	Resistant	–	–	PF	Yes^c^
XW‐3N‐9	K120M	Homo‐/hemi‐	Resistant	↑	–	NF	N/A
XW‐3N‐14	A161P R249S	Hetero‐ Hetero‐	Mixed	↑	↑	PF NF	N/A Yes^3^, ^4^
XW‐3N‐15	A161G L194R	Hetero‐ Hetero‐	Mixed	↑	↑	PF NF	No^d^ N/A
XN‐3N‐105	R273L	Hetero‐	Resistant	–	–	NF	Yes^3^, ^4^
XN‐3N‐110	intron 5 (SA)	Homo‐/hemi‐	Resistant	–	–	N/A	N/A
XN‐3N‐117	V157F	Hetero‐	Resistant	–	–	NF	Some^c^
XN‐3N‐120	V157F	Hetero‐	Resistant	–	–	NF	Some^c^
**B) Established in parallel to a Nutlin‐3a counter‐screen**							
XW‐3N‐29	intron 3 (SA)	Homo‐/hemi‐	Resistant	–	–	N/A	N/A
XW‐3N‐37	R282W	Homo‐/hemi‐	Resistant	–	–	NF	Some^c^, no^d^
XW‐3N‐43	R248W	Hetero‐	Resistant	↑	–	NF	Yes^c^
XW‐3N‐54	H178Q H179P	Hetero‐ Hetero‐	Resistant	–	–	NF PF	N/A N/A
XW‐3N‐55	R175L	Homo‐/hemi‐	Resistant	↑	↑	PF	No^c^
XW‐3N‐59	R158L	Homo‐/hemi‐	Resistant	–	–	NF	Some^c^
XN‐3N‐136	G245R	Homo‐/hemi‐	Resistant	–	–	NF	Yes^c^
XN‐3N‐137	G244A	Hetero‐	Resistant	–	–	NF	N/A
	R249T	Hetero‐				NF	N/A
XN‐3N‐140	C277F	Hetero‐	Resistant	–	–	NF	N/A
XN‐3N‐141	C277Y	Hetero‐	Sensitive; rapid recovery	↑	↑	NF	Yes^c^, no^d^
XN‐3N‐151	c124, frameshift	Homo‐/hemi‐	Resistant	–	–	N/A	N/A
XN‐3N‐156	R273S	Hetero‐	Resistant	–	–	NF	Yes^e^
XN‐3N‐157	intron 8 (SA)	Homo‐/hemi‐	Resistant	–	–	N/A	N/A

a XW = Xpa‐WT; XN = Xpa‐Null; 3N = 3‐NBA‐treated.

b The overall transactivation activity of the mutant in a yeast functional assay published by Kato *et al*.^d^ (NF = non‐functional, PF = partially functional, F = functional, N/A = not assessed).

c Dearth *et al*.^27^

d Marutani *et al*.^28^

e Mitsumoto *et al*.^29^

### Expression and activation of p53 in HUFs following treatment with Nutlin‐3a

**Figure 2:**
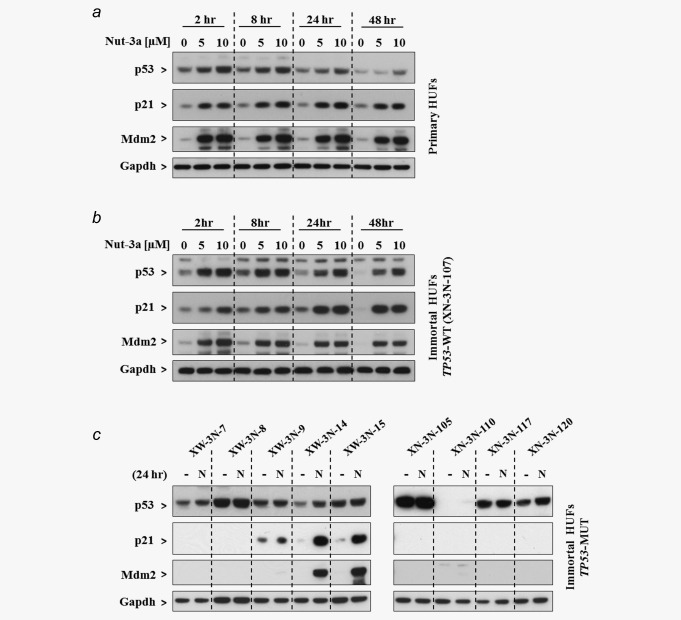
Induction of p53, p21 and Mdm2 in primary and immortal HUFs treated with Nutlin‐3a. Protein expression of p53, p21 and Mdm2 was assessed by Western blotting of whole cell lysates. Gapdh served as a loading control. (*a*) Primary or (*b*) immortal HUFs with WT *TP53* (clone XN‐3N‐107) were treated for 2, 8, 24 or 48 hr ± 5 or 10 μM Nutlin‐3a. (*c*) Immortal HUFs with mutated *TP53* were treated without (−) or with (N) 10 μM Nutlin‐3a for 24 hr.

We next assessed the ability of Nutlin‐3a to stabilise and activate p53 in HUFs. Initially, primary HUFs with WT *TP53* were treated with 5 or 10 µM Nutlin‐3a for 2–48 hr, and the expression of p53 and its downstream targets p21 and Mdm2 was assessed by Western blotting (Fig. 2 *a*). p53 expression was maximally stabilised after 2‐hr treatment with Nutlin‐3a, with a similar level of expression maintained after 8 hr. Thereafter, Nutlin‐3a‐induced expression of p53 decreased; by 48 hr p53 expression had nearly returned to the basal level. p21 and Mdm2 were also induced after only 2‐hr treatment with Nutlin‐3a, and their expression was maintained up to 48 hr. Maximal induction of p21 was observed at 8–24 hr post‐treatment, while Mdm2 expression peaked at 2 hr. Similar results were obtained for Xpa‐WT and Xpa‐Null HUFs (data not shown).

The impact of Nutlin‐3a on p53 signalling was further evaluated in immortal HUFs (clone XN‐3N‐107) with WT *TP53* (Fig. 2 *b*). For the most part Nutlin‐3a‐induced p53 stabilisation, as well as the expression of p21 and Mdm2, in the immortal HUFs were similar temporally to those observed in primary HUFs. However, p53 stabilisation and p21 induction at later timepoints (24 and 48 hr) appeared to be more robust in immortal HUFs than in primary HUFs. Similar responses were found in another immortal HUF cell line with WT *TP53*, XN‐C‐327 (data not shown).

Next, p53 expression and activation was examined in nine *TP53*‐mutated immortal HUF cell lines treated with and without 10 µM Nutlin‐3a for 24 hr (Fig. 2 *c* and Table 1). In most clones, p53 expression was stabilised even in the absence of Nutlin‐3a, as previously reported for HUFs containing missense mutations in *TP53*;^13^ Nutlin‐3a treatment did not induce a further increase in expression. However, some increase of p53 expression upon Nutlin‐3a treatment was observed in clone XW‐3N‐14. No p53 expression was detected in clone XN‐3N‐110, in agreement with the fact that this clone is mutated at the splice acceptor site for intron 5. Most *TP53*‐mutated HUFs were not able to induce p21 or Mdm2 expression following Nutlin‐3a treatment; however p21 was induced in clones XW‐3N‐9, XW‐3N‐14 and XW‐3N‐15, and Mdm2 was induced in clones XW‐3N‐14 and XW‐3N‐15.

### *TP53* mutations in HUF cell lines with a mixed response to Nutlin‐3a

**Figure 3:**
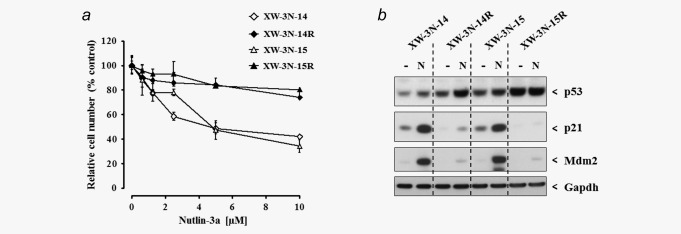
HUF cell lines exhibiting a mixed response to Nutlin‐3a contain a Nutlin‐3a‐resistant subpopulation. Growth and p53 activation in response to treatment with Nutlin‐3a was compared in parental (XW‐3N‐14 and −15) and Nutlin‐3a‐resistant (XW‐3N‐14R and −15R) cells. (*a*) Relative cell number following 5 days treatment with Nutlin‐3a was determined using crystal violet staining and mean values are shown as % of control ± SD of five replicate wells. Data are representative of at least two experiments. (*b*) Expression of p53, p21 and Mdm2 ± 10 μM Nutlin‐3a (24 hr) was assessed by Western blotting. Gapdh served as a loading control.

To explore further the mixed growth response of cell lines XW‐3N‐14 and XW‐3N‐15 to Nutlin‐3a, Nutlin‐3a‐resistant cells from the cultures that had been treated with Nutlin‐3a for five days were expanded. The resistant populations (designated XW‐3N‐14R and XW‐3N‐15R) were expanded for two to three passages and subsequently compared to the parental lines for their response to Nutlin‐3a. Following 5 days of retreatment with Nutlin‐3a, the parental cell lines again exhibited partial sensitivity to growth inhibition, whereas XW‐3N‐14R and XW‐3N‐15R were almost completely resistant to retreatment (Fig. 3 *a*). Additionally, while p21 and Mdm2 expression was induced in the parental cell lines following 24‐hr treatment with Nutlin‐3a, little to no expression was detected in the Nutlin‐3a‐resistant sublines (Fig. 3 *b*). These data indicate that Nutlin‐3a‐resistance is dependent on loss of WT p53 transcriptional activity.

In order to determine whether the Nutlin‐3a‐resistance of XW‐3N‐14R and XW‐3N‐15R could be explained by additional or altered *TP53* mutation(s) in a subpopulation of the parental line, single cell clones were generated for XW‐3N‐14/‐14R and XW‐3N‐15/‐15R. *TP53* (exons 4–9) was then sequenced from each single cell clone (Supporting Information Table 2). Note that in the original parental populations, XW‐3N‐14 harboured A161P and R249S mutations while XW‐3N‐15 carried A161G and L194R mutations.

All single cell clones isolated from XW‐3N‐14 were mutated at codon 249 (Supporting Information Table 2). However, only two of six clones were mutated at codon 161; the other four clones were wild‐type at this site. Interestingly, all of the single cell clones isolated from the Nutlin‐3a‐resistant line XW‐3N‐14R were mutated at both 249 and 161. This indicates that cells harbouring only the R249S mutant were sensitive to Nutlin‐3a and the additional A161P mutation was required for resistance to Nutlin‐3a. It is unclear whether the mutations occur on separate *TP53* alleles or whether both mutations are on the same allele with the second allele remaining WT.

The single cell clones isolated from XW‐3N‐15 all contained heterozygous mutations at both codons 161 and 194 (Supporting Information Table 2). Likewise, mutations were detected at codons 161 and 194 in single cell clones from the Nutlin‐3a‐resistant line XW‐3N‐15R. However, the mutations in five out of six single cell clones from the Nutlin‐3a‐resistant line were homo‐/hemizygous. Most likely the two mutations occurred on the same allele, and the second (WT) allele, retained by the Nutlin‐3a‐sensitive cells in the parental line, was lost in the Nutlin‐3a‐resistant cells. It is unclear whether Nutlin‐3a induced loss of the WT allele (*i.e*. LOH) or whether Nutlin‐3a‐treatment selected for a small population of the parental cell line that had already undergone LOH. Regardless, loss of the WT allele appears to be required for Nutlin‐3a resistance in this mutant.

### *TP53*‐mutated clones are selectively resistant to a Nutlin‐3a counter‐screen during the HIMA

**Figure 4:**
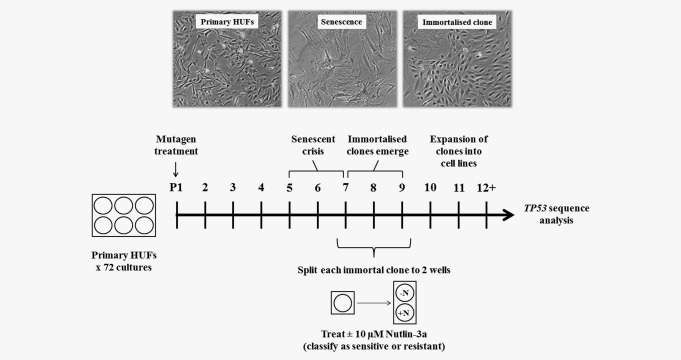
Schematic of the HUF immortalisation assay (HIMA) including a counter‐screen with Nutlin‐3a. Primary HUFs were treated with 1 µM 3‐NBA and serially passaged at 80 to 90% confluence until senescent crisis (∼P5–P7). As immortalised clones emerged (∼P7–P9), each culture was split to two wells and treated ±10 µM Nutlin‐3a for 5 days. Following treatment, clones were classified as sensitive or resistant to Nutlin‐3a. Untreated cells were continuously cultured until completion of immortalisation (P12+) and then DNA sequenced to identify *TP53* mutations. “P” refers to passage number.

The results of the initial stage of this study indicated that treatment with Nutlin‐3a for 5 days could be used to distinguish between immortal HUF clones containing WT or MUT *TP53*. We next sought to determine whether a counter‐screen with Nutlin‐3a could be integrated as part of the HIMA. Therefore, a HIMA was conducted according to the standard protocol with a Nutlin‐3a‐counter‐screen performed in parallel (Fig. 4). Seventy‐two cultures of primary HUFs (36 Xpa‐WT, 36 Xpa‐Null) were treated with 3‐NBA to induce mutations and then passaged until senescent crisis. As immortalised clones emerged from the cultures and exhibited robust proliferation (passage 6–10; 1–3 months), each was treated ±10 µM Nutlin‐3a for 5 days. The sensitivity or resistance of each clone was assessed by visual inspection under the microscope. Following treatment, Nutlin‐3a‐containing media was replaced with normal media for all sensitive clones, and treated cultures were observed for growth for an additional 7 days. The response of each clone is listed in Supporting Information Table 3. The parallel, untreated (−Nutlin‐3a) culture for each clone was continuously passaged until immortalisation was completed.

Eleven clones (XW‐3N‐29, −37, −43, −55, −59; XN‐3N‐136, −137, −140, −151, −156, −157) were completely resistant to Nutlin‐3a. One clone (XW‐3N‐54) exhibited a mixed response to Nutlin‐3a treatment at passage 9 but was completely resistant when retested at passage 13. Interestingly, one clone (XN‐3N‐141) was very sensitive to Nutlin‐3a but exhibited robust recovery 2 to 3 days after Nutlin‐3a‐containing media was replaced with normal growth media. The response of XN‐3N‐141 did not change when the clone was retested at a later passage. After 7 days in normal media post‐treatment, varying degrees of recovery were observed for some other Nutlin‐3a‐sensitive clones while most clones remained completely growth arrested.

Upon completion of immortalisation (≥12 passages), *TP53* (exons 4–9) was sequenced from each clone. *TP53* mutations were identified in all 12 clones that were resistant to Nutlin‐3a (Table 1), whereas only one Nutlin‐3a‐sensitive clone, XN‐3N‐141, harboured a *TP53* mutation.

**Figure 5:**
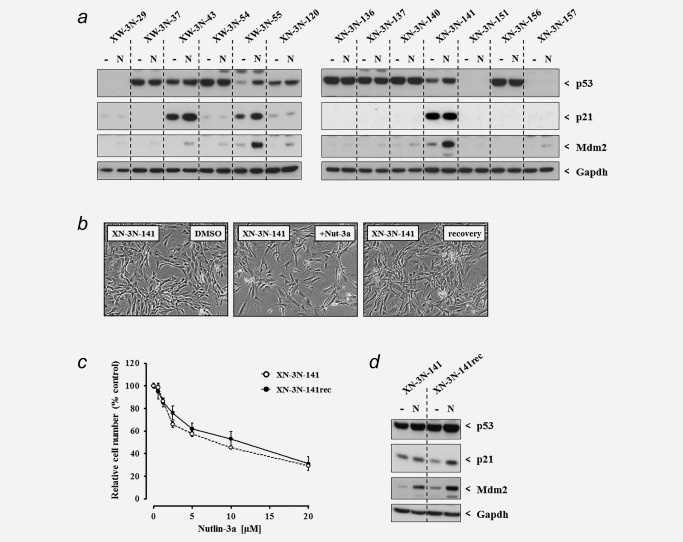
Evaluation of the response to Nutlin‐3a in *TP53*‐mutant HUF cell lines established in parallel to a Nutlin‐3a counter‐screen. (*a*) Protein expression of p53, p21 and Mdm2 was assessed by Western blotting whole cell lysates of HUFs treated without (−) or with (N) 10 μM Nutlin‐3a for 24 hr. Gapdh served as a loading control. (*b*–*d*) Growth and p53 activation in response to treatment with Nutlin‐3a was compared in the Nutlin‐3a‐sensitive *TP53*‐mutant clone XN‐3N‐141 and subline XN‐3N‐141rec (generated following an initial 5‐day treatment with Nutlin‐3a). (*b*) Photomicrographs (100×) of untreated (0.1% DMSO) cells, cells treated with 10 μM Nutlin‐3a for 5 days (+Nut‐3a) and cells allowed to recover for 2 days following Nutlin‐3a treatment (recovery). (*c*) relative cell number following 5 days treatment with Nutlin‐3a was determined using crystal violet staining and mean values are shown as % of control ± SD of five replicate wells. Data are representative of at least two experiments. (*d*) Expression of p53, p21 and Mdm2 ± 10 μM Nutlin‐3a (24 hr) was assessed by Western blotting. Gapdh served as a loading control.

Finally, each clone (≥12 passages) harbouring a *TP53* mutation was treated ± Nutlin‐3a for 24 hr to assess the expression of p53, p21 and Mdm2 by Western blotting (Table 1 and Fig. 5 *a*). A high level of constitutive p53 expression was observed for eight of the HUF cell lines carrying missense mutations, which was not increased by Nutlin‐3a treatment. With the exception of XN‐3N‐43 (R248W), these clones were incapable of inducing p21 or Mdm2 expression in response to Nutlin‐3a. XN‐3N‐43 exhibited constitutive p21 expression, but was unable to induce Mdm2. Two clones, XW‐3N‐55 (R175L) and XN‐3N‐141 (C277Y), expressed a low basal level of p53 that increased following Nutlin‐3a treatment and were the only mutants capable of inducing both p21 and Mdm2 in response to Nutlin‐3a. Finally, the three clones harbouring mutations at intronic splice sites exhibited no p53 expression and weak‐to‐no induction of p21 or Mdm2.

### Reversible Nutlin‐3a sensitivity of a TP53‐mutated clone (XN‐3N‐141)

As described above, clone XN‐3N‐141 (C277Y) was sensitive to Nutlin‐3a, despite harbouring a *TP53* mutation. Five days of treatment with Nutlin‐3a caused the cells to arrest, but they remained morphologically normal and were not enlarged (Fig. 5 *b*), in contrast to the morphological changes observed in Nutlin‐3a‐sensitive *TP53*‐WT clones (compare with Fig. 1 *b*). However, once Nutlin‐3a was removed and replaced with normal media, XN‐3N‐141 cell growth recovered within 2 to 3 days (Fig. 5 *b*). This rapid recovery was not observed in any of the clones containing WT‐*TP53*. The cells that recovered from Nutlin‐3a‐treatment (XN‐3N‐141rec) were expanded and retested. Both the parental line, XN‐3N‐141rec, and the Nutlin‐3a‐selected line, XN‐3N‐141R, arrested when treated with Nutlin‐3a for 5 days (Fig. 5 *c*) and both exhibited p21 and Mdm2 induction after Nutlin‐3a treatment for 24 hr (Fig. 5 *d*).

## Discussion

The HIMA is a unique tool in the current arsenal of mutagenesis assays, enabling the generation and selection of mutations in a human cancer‐related gene (*i.e. TP53*) within mammalian cells. Mutation or loss of *TP53* is a key mechanism in the immortalisation of HUFs. However, because other genetic alterations also enable immortalisation of HUFs, the HIMA is not selective only for *TP53*‐mutated cells. In an attempt to improve the selectivity of the HIMA, we examined whether the Mdm2‐inhibitor Nutlin‐3a could be used to identify clones harbouring *TP53* mutations. In this study, over 86 immortal HUF clones were tested with Nutlin‐3a, including 22 with identified *TP53* mutations. We showed that Nutlin‐3a stabilised and activated WT p53, leading to growth suppression of all primary and immortal HUF cell lines with WT *TP53*. On the other hand, immortal HUFs harbouring *TP53* mutations were, for the most part, resistant to Nutlin‐3a‐induced growth inhibition and p53 activation. Only 1/22 clones (XN‐3N‐141) with mutated *TP53* was sensitive to Nutlin‐3a; however, this clone recovered rapidly upon removal of Nutlin‐3a in a manner that was not observed for any of the *TP53*‐WT clones. Importantly, we found that when immortal HUF clones were treated with Nutlin‐3a soon after their emergence from senescent cultures during an immortalisation assay, resistance to Nutlin‐3a was highly predictive of the presence of *TP53* mutation(s). Therefore, we propose that future HIMAs may include a Nutlin‐3a counter‐screen, whereby only Nutlin‐3a‐resistant clones are assessed for *TP53* mutations and Nutlin‐3a‐sensitive clones are discarded.

Although most *TP53*‐mutated clones were completely resistant to Nutlin‐3a‐induced growth suppression, two clones exhibited a mixed response to Nutlin‐3a treatment (*i.e*. contained both sensitive and resistant cells). In these cases cells retaining one WT *TP53* allele, in addition to the mutated allele, remained Nutlin‐3a‐sensitive, whereas cells that acquired a second mutation or lost the WT *TP53* allele were Nutlin‐3a‐resistant. As most heterozygously‐mutated clones were completely resistant to Nutlin‐3a, the Nutlin‐3a‐sensitivity of some cells harbouring both mutant and WT p53 may be explained by a mutant that retains some WT functionality or by a non‐functional mutant that fails to act dominant‐negatively against the WT protein. Indeed, while p21 and Mdm2 expression were efficiently induced by Nutlin‐3a in clones XW‐3N‐14 and XW‐3N‐15 (which were Nutlin‐3a‐sensitive and retained a WT *TP53* allele), the Nutlin‐3a‐resistant sublines XW‐3N‐14R (which acquired a second mutation) and XW‐3N‐15R (which lost the WT *TP53* allele) were unable to induce expression of the p53 targets p21 and Mdm2 following Nutlin‐3a treatment. Although the *TP53* mutations in the Nutlin‐3a‐sensitive clones clearly permitted these cells to escape from senescence and to immortalise, perhaps the level of WT p53 stabilisation induced by Nutlin‐3a overwhelmed the mutant p53 protein to an extent not observed under normal growth conditions.

Interestingly, the one clone (XN‐3N‐141) that exhibited reversible sensitivity to Nutlin‐3a harboured a mutation at the same *TP53* codon (277) mutated in another clone that was completely resistant to Nutlin‐3a (XN‐3N‐140); in both cases the mutation was heterozygous. In XN‐3N‐141 the mutation converts C277 to Y277, while in XN‐3N‐140 C277 is converted to F277. The ability to induce p21 and Mdm2 expression was lost in the C277F mutant, but retained in the C277Y mutant. This suggests that the specific amino acid change generated by a mutation plays an important part both in the response of a clone to Nutlin‐3a and the ability of the mutant protein to exert dominant‐negative effects over wild‐type p53. Further studies to examine additional HUF cell lines could be performed to determine how frequently, or rarely, *TP53*‐mutated clones exhibit sensitivity to Nutlin‐3a. The HUF clones generated in previous HIMAs represent over 100 unique mutations in *TP53*, including several different mutations at the same codon. The functional impact of these mutations is diverse (*i.e*. silent, functional, partially‐functional and non‐functional) and could be investigated by screening these clones for their response to Nutlin‐3a.

Notably, the majority of *TP53*‐mutated clones were identified as resistant to Nutlin‐3a within 2.5 months of initiating the HIMA (Supporting Information Table 3). Thus, a Nutlin‐3a counter‐screen could be used to identify mutants within a cut‐off of 2.5 months. Limiting the detection of mutant (Nutlin‐3a‐resistant) clones to a 2.5 month period and discarding WT (Nutlin‐3a‐sensitive) cultures would greatly reduce the labour of the assay. The current study suggests that few mutants would be missed by this approach.

One potential limitation of the Nutlin‐3a counter‐screen would be the likely Nutlin‐3a‐sensitivity of clones carrying silent *TP53* mutations, since these clones would retain WT p53 functional activity. However, silent mutations have been observed infrequently in previous HIMAs and were not found in the current study.^5^ It is also unclear whether other genetic alterations, particularly those affecting p53 pathway components, would impact the response of an immortal clone to Nutlin‐3a. Previous studies indicated that MEFs lacking p19 and p21 are still sensitive to Nutlin‐3a, although loss of p21 appears to hinder the ability of Nutlin‐3a to induce permanent cell cycle arrest.^22^ Another study showed that loss of p19 potentiated Nutlin‐3a‐induced growth inhibition of neuroblastoma cells.^30^ Further, MDM2 overexpression reportedly enhances cell sensitivity to Nutlin‐3a.^31^ We predict that the unique nature of each *TP53* mutant (*e.g*. transactivational capability or dominant negative effects), in combination with the presence or loss of the WT *TP53* allele, will be the main determinant of Nutlin‐3a‐resistance or sensitivity.

Although Nutlin‐3a is considered to be a non‐genotoxic activator of p53, two studies have shown that long‐term exposure to Nutlin‐3a can induce *TP53* mutations. In the first report, SJSA‐1 osteosarcoma cells were exposed to four cycles of treatment with 10 µM Nutlin‐3a for 72 hr followed by a recovery period.^32^ Five unique *TP53* mutations not found in the parental cell line were identified in subclones. In a separate study, six different cancer cell lines were exposed continuously to Nutlin‐3a for up to 14 passages.^33^ Here, 28 out of 35 Nutlin‐3a‐adapted sublines contained *TP53* mutations. This includes 8 mutants that were generated from a single cell clone of the *TP53*‐WT cell line UKF‐NB‐3, indicating that Nutlin‐3a did not merely select for rare mutants already present in the cell line. These data suggest that spontaneous *TP53* mutations may be selected for under the stress of Nutlin‐3a treatment. The authors postulated that perhaps deficiencies in DNA repair or replication fidelity in cancer cells can enhance the induction of spontaneous mutations. The potential for Nutlin‐3a to induce *TP53* mutations would not be an issue when it is used to counter‐screen immortal HUFs in the manner proposed here. Resistance or sensitivity to short‐term (5 days) Nutlin‐3a treatment simply serves to identify *TP53*‐WT and *TP53*‐MUT immortal clones. *TP53* should then be sequenced from cultures that were not exposed to Nutlin‐3a, although it may also be useful to sequence *TP53* from Nutlin‐3a‐resistant cells expanded following Nutlin‐3a treatment in some cases, as done in this study.

In conclusion, this proof‐of‐concept study has shown that resistance to the Mdm2‐inhibitor Nutlin‐3a is a common feature of immortal HUFs harbouring mutated *TP53*; thus, a Nutlin‐3a counter‐screen during the HIMA can be used to select *for TP53*‐mutated cells and select *against TP53*‐WT cells soon after the immortal clones become established. In this way, Nutlin‐3a‐sensitive clones can be discarded and *TP53* mutations assessed only in Nutlin‐3a‐resistant clones, improving the efficiency of the assay. More assays can be performed, more clones screened, and more *TP53* mutations generated, thereby enhancing the study of mutagenesis of a gene with so many possible inactivating mutations.

## Supporting information

Supporting Information
