# Supplementary material for: Nutlin‐3a selects for cells harbouring TP 53 mutations
Source: Int J Cancer. 2016 Nov 22;140(4):877–87. doi: 10.1002/ijc.30504 (PMC5215675; doi:10.1002/ijc.30504)
Supplement: Supplementary file 1 — Supporting Information [file IJC-140-877-s001.doc]

**Supplementary Table I. Summary of immortal HUFs used in this study.**

**Supplementary Table II. *TP53* mutations detected in single cell clones derived from parental and Nutlin-3a-resistant HUF sublines**

**Supplementary Table III. The response of immortal HUF clones to a Nutlin-3a counter-screen conducted in parallel to a standard HIMA.** 72 cultures of primary HUFs were treated with 1 µM 3-NBA for 48 hr and serially passaged until immortalisation. Immortal clones emerged from each culture between 30–84 days (or 7–10 passages) after initiating the assay. Immortal clones were treated ± 10 µM Nutlin-3a for 5 days, qualitatively assessed for growth by visual observation under the microscope and classified as sensitive or resistant to Nutlin-3a. Note that three cultures did not develop immortalised clones.

**Reference for Supplementary Material:**

1. Kucab JE, Zwart EP, van Steeg H, et al. (2016) TP53 and lacZ mutagenesis induced by 3-nitrobenzanthrone in Xpa-deficient human TP53 knock-in mouse embryo fibroblasts. *DNA Repair (Amst)*, 39, 21-33.
